# Adipocytes and intestinal epithelium dysfunctions linking obesity to inflammation induced by high glycemic index pellet-diet in *Wistar* rats

**DOI:** 10.1042/BSR20180304

**Published:** 2018-06-27

**Authors:** Anna Beatriz Santana Luz, Júlia Braga dos Santos Figueredo, Bianca Damásio Pereira Dantas Salviano, Ana Júlia Felipe Camelo Aguiar, Luiza Gabriella Soares Dantas Pinheiro, Matheus Felipe Dantas Krause, Christina da Silva Camillo, Fernando Vagner Lobo Ladd, Raul Hernandes Bortolin, Vivian Nogueira Silbiger, Bruna Leal Lima Maciel, Ana Heloneida de Araújo Morais

**Affiliations:** 1 Nutrition Post Graduate Program, Center for Health Sciences, Federal University of Rio Grande do Norte, Natal, RN 59078-970, Brazil; 2 Biochemistry Post Graduate Program, Biosciences Center, Federal University of Rio Grande do Norte, Natal, RN 59078-970, Brazil; 3 Nutrition Course, Center for Health Sciences, Federal University of Rio Grande do Norte, Natal, RN 59078-970, Brazil; 4 Medicine Course, Center for Health Sciences, Federal University of Rio Grande do Norte, Natal, RN 59078-970, Brazil; 5 Department of Morphology, Biosciences Center, Federal University of Rio Grande do Norte, Natal, RN 59078-970, Brazil; 6 Pharmaceutical Sciences Post Graduate Program, Centre for Helth Sciences, Federal University of Rio Grande do Norte, Natal, RN 59078-970, Brazil; 7 Clinical and Toxicological Analysis, Center for Health Sciences, Federal University of Rio Grande do Norte, Natal, RN 59078-970, Brazil; 8 Department of Nutrition, Center for Health Sciences, Federal University of Rio Grande do Norte, Natal, RN 59078-970, Brazil

**Keywords:** adiposity, animal feed, dietary sucrose, TNF-α

## Abstract

We investigated the inflammatory effect of a pellet-diet with high glycemic index and load (HGLI) on the histological organization of adipocytes, intestinal epithelium, and fat in liver and pancreas in adult male *Wistar* rats. Two groups (*n*=10) received for 17 weeks: (1) HGLI diet or (2) Standard diet (Labina®). Histological analyses of adipose tissue, jejunum, liver, and pancreas were performed. Stereology analysis, visceral adiposity index, gene expression, and immunohistochemistry of tumor necrosis factor-α (TNF-α) in visceral adipose tissue and plasma TNF-α were also assessed. The HGLI diet-induced hypertrophy of adipocytes with adipocyte volume density equal to 97.0%, cross-sectional area of adipocytes equivalent to 1387 µm² and a total volume of adipocytes of 6.97 cm³ an elevation of 8%, 25%, and 58%, respectively. Furthermore, the HGLI diet increased liver and pancreatic fat deposition, altered and inflamed the intestinal epithelia, and increased TNF-α gene expression (*P*=0.014) with a positive immunostaining in visceral adipose tissue and high plasma TNF-α in comparison with standard diet. The results suggest that this diet was able to generate changes commonly caused to solid diets with high fat or fructose-rich beverages. To the best of our knowledge, this is the first report in the literature concerning the properties of low-cost, sucrose-rich pellet-diet presenting high glycemic index and high glycemic load efficient on the development of obesity complications in *Wistar* rats that were subjected to diet-induced obesity. Therefore, the HGLI pellet-diet may be considered an effective tool to be used by the scientific community in experimental research.

## Introduction

Obesity is characterized by the accumulation of adipose tissue, independently of age, sex, and height [1,2] and is directly related to inflammation and various chronic non-communicable diseases, influencing body composition [3]. The eating pattern of modern western society has been the leading cause of obesity in the world and is represented by excessive consumption of processed foods, added sugars, and refined grains. This consumption, associated with reduced intake of fruits, vegetables and fish, results in a nutritionally unbalanced diet with high energy content, insufficient supply of fiber, vitamins and minerals, as presents a high glycemic index [4].

Good dietary choices represent effective means for glycemic and body weight control and are considered a valuable tool in the primary and secondary prevention of disorders such as obesity, Type 2 diabetes mellitus, systemic arterial hypertension, dyslipidemia, metabolic syndrome, inflammatory diseases, and cardiovascular diseases [5]. However, due to ethical concerns with the long-term application of nutritionally unbalanced diets in humans, aiming to analyze their effects [6], many researches are developed on rodents. These experimental diets reproduce the basic characteristics of human diets to induce metabolic, body, and biochemical changes [7,8].

Such diets contain highly caloric and tasty ingredients, being similar to the diets usually consumed by the human population. As a result, they are able to generate hyperphagia and consequently weight gain, being mostly related to the increase in adipose tissue in laboratory animals [9,10]. However, there is little or no information for these diets, about the glycemic index or the glycemic load [11]. The glycemic index of foods has received great attention in recent years and has been recommended as a criterion for the choice of carbohydrate source foods [1]. In this context, it is also important to highlight the glycemic load, which is the product of the glycemic index and the total available carbohydrates present in a given quantity of food [2].

There have been no reports of effects, especially associated with inflammatory mediated alterations involving tumor necrosis factor-α (TNF-α), of a high glycemic index and load pellet-diets in male *Wistar* rats. What has been reported is related to the use of beverages rich in fructose and/or sucrose, either alone or in association with diets, but not specifically pellet-diets [12–15]. Studies dealing with pellet-diets similar to the food pattern of modern western society, with an emphasis on inflammatory parameters, were performed in mice [16–18] and, unsuccessfully, in *Wistar* rats [19].

Interestingly, this lineage is susceptible to diet-induced obesity (DIO) and insulin resistance with individual variations [20], since it comes from outbred rat’s strain [21]. However, depending on the type of DIO, *Wistar* rats present completely different obesity phenotypes, as observed in the study by Bortolin et al. [12], in which they emphasize the importance of diet selection for this purpose.

Thus, we investigated the effect of a pellet-diet with high glycemic index and load (HGLI) on the histological organization of adipocytes and intestinal epithelium; ectopic accumulation of fat in liver and pancreas; stereological aspects; the index of visceral adiposity; mRNA expression, immunostaining and plasma concentrations of TNF-α in adult male *Wistar* rats. The results obtained are novel and reveal that the HGLI diet can be safely used as another tool in experimental research, given the information generated here regarding its effects in *Wistar* rats.

## Materials and methods

### Animals and experiment design

Male *Wistar* rats (*n*=10) were used, weighing 320–380 g from the Potiguar University (UnP), Natal-RN. All experiments were developed according to the Guide for the Care and Use of Laboratory Animals [22] and approved by the Committee on Ethics in the Use of Animals (CEUA-UnP) under protocol n° 012/2015.

Animals were randomly assigned and equally distributed (five rats per group) into two experimental groups, which correspond to the animals submitted to the experimental diet (HGLI) and control group (standard), submitted to the commercial Labina® diet. The experimental period was 17 weeks, time required for the diagnosis of obesity, according to Lee index proposed by Bernardis [23], and classified by Novelli (>0.300 g/cm³) [24]. The rats stayed in individually ventilated cages, in the standard light condition (12/12-h light/dark), temperature (23–25°C), and humidity (50 ± 5%), with water and food *ad libitum*.

### Diets

The diets used in the experiments were the Labina® diet (Paulínia, São Paulo, Brazil), offered to the control group, and the HGLI diet, offered to the experimental group, which presented 315.26 kcal, 21% of proteins, 4% of lipids, and 48% of carbohydrates [25]. This diet was characterized by a high glycemic index and glycemic load, with values of 77.6 and 38.8 respectively. The determination of these parameters was performed according to Aguiar et al. [25], using established methodologies for the evaluation of human diets.

For the preparation of 100 g of the HGLI diet, 45.2 g of the Labina® ration was grounded using a food processor, adding 9.6 g of refined sugar and 45.2 ml of condensed milk, followed by manual homogenization. Then, the diet was cast in the form of cylinders, which were baked in a preheated oven at 180°C, for approximately 40 min, according to methodology previously described by our group [25]. Both diets were offered *ad libitum*. Condensed milk and refined sugar were purchased commercially, with the same batch and brand for the entire experimental period.

### Collection of blood, adipose tissue, and other organs

After 17 weeks of study, with confirmation of obesity by the Lee index, animals were fasted for 8–12 h and then anesthetized with 250 mg of tiletamine hydrochloride and 250 mg of zolazepam hydrochloride for the collection of whole blood through the hepatic portal vein. The animals were killed and the small intestine (jejunum), visceral adipose tissue (perirenal, retroperitoneal, and epididymal), liver, and pancreas were collected for further analysis.

### Histological examination of the small intestine, visceral adipose tissue, liver, and pancreas

The analysis was performed according to Martins et al. [26] with some modifications. Sections of the tissues (3–4 μm) were stained with hematoxylin and eosin. For the histological analysis, the diagnostic reading of the slides was performed with emphasis on the histological organization of the jejunum and visceral adipose tissue. In addition, the presence of adipocytes in the hepatic and pancreatic tissues of the studied groups was investigated. The evaluation was done using a CX21 microscope (Olympus, Shinjuku, Tokyo, Japan). The images were captured using a DS-Ri1 digital camera (Nikon, Edgewood, New York, U.S.A.) coupled to an Eclipse Ni (Nikon, Edgewood, New York, U.S.A.) (200×) microscope.

### Stereological analysis of visceral adipose tissue

The morphoquantitative parameters (volume density, mean cross-sectional area, and total volume) of the adipocytes present in the visceral adipose tissue (perirenal, retroperitoneal, and epididymal) were made considering the histological sections and fields of view, sampled by a uniform, systematic, and random way based in Gundersen et al. [27] and the mean cross-sectional area of the adipocytes was evaluated by stereology as the ratio between the volume density of adipocytes and twice the numerical density per area of adipocytes [28]. Volume density was estimated by point counting on a test system, and numerical density was estimated as the ratio between the number of adipocytes counted into a frame and the test area of the frame. And the total volume was obtained multiplying the volume density of adipocytes by the sample weight [29].

### Visceral adiposity index

For the adiposity index, the visceral adipose tissue of the animals was individually weighed on a precision scale. The sum of the three compartments of adipose tissue was considered as total visceral fat. The visceral adiposity index was calculated by adapting the formula used by Leopoldo et al. [30], replacing total body fat with total visceral fat:

VA=(Total visceral fat÷Final body weight)×100

### Expression of mRNA, immunohistochemistry, and plasma concentrations of TNF-α

Adipose tissue of the animals, previously stored at −80°C, was pulverized with liquid nitrogen and the total RNA was extracted using the commercial Trizol™ Plus RNA Purification Kit (Thermo Fisher Scientific, Waltham, Massachusetts, U.S.A.), following the manufacturer’s instructions. Quantification was obtained using the NanoDrop ND-2000 UV-Vis spectrophotometer (Thermo Fisher Scientific, Wilmington, Delaware, U.S.A.). cDNA synthesis was performed starting from 500 ng of total RNA through the High-Capacity cDNA Transcription Kit set (Thermo Fisher Scientific, Waltham, Massachusetts, U.S.A.), according to the manufacturer’s instructions, using a MyCycler™ thermal cycler (Bio-Rad Laboratories, Hercules, California, U.S.A.). cDNA was obtained in a final volume of 20 μl and stored at −20°C until used for the RT-qPCR expression assays.

RT-qPCR was performed using the TaqMan® RT-PCR amplification system (Applied Biosystems, Foster City, California, U.S.A.) for the *TNF-α* gene (LOC103694, Rn01525859_g1) and *GAPDH* (Rn01775763_g1) (Thermo Fisher Scientific, Waltham, Massachusetts, U.S.A.), used as an endogenous control gene. PCR assays were performed using the ABI Prism 7500 FAST equipment (Applied Biosystems, Foster City, Calif., U.S.A.). The relative expression was calculated using the 2^−ΔΔ*C*^_t_ method [31] and the results are presented as fold change versus mean values of the control group normalized for the endogenous *GAPDH* gene.

For immunohistochemistry, procedures were performed according to Khan et al. [32], with some modifications. The material was dehydrated and paraffin embedded, with sections of 3 μm thickness in a paraffin rotary microtome and subsequent assembly in gelatinized slides. These were then deparaffinized and hydrated. Antigen retrieval was performed using a heating plate for 20 min to reach 80°C. Between each step, five 5-min wash cycles in 0.1 M sodium phosphate buffer, pH 7.4, were used.

For the detection of TNF-α in the adipose tissue, the Rabbit Specific HRP/DAB kit (ABC) (Abcam, Cambridge, U.K.) was used according to the manufacturer’s instructions, and the TNF-α rabbit polyclonal primary antibody (1:100 dilution) (Abcam, Cambridge, U.K.) was incubated overnight. For analysis, images of adipose tissue after immunohistochemistry were captured using a DS-Ri1 digital camera (Nikon, Edgewood, New York, U.S.A.) coupled to an Eclipse Ni (Nikon, Edgewood, New York, U.S.A.) (100×) microscope.

The intensity of TNF-α was measured semiquantitatively and automatically by means of the determination of the optical density in diaminobenzidine staining (DAB) images since the optical density is proportional to the staining concentration. The evaluation was based on Image J software (version 1.51), using a plugin known as IHC profiler [33].

The DAB staining images were analyzed pixel by pixel by this plugin and the scoring was given according to a grade, which consisted of the following variation: negative (0), low positive (+1), positive (+2), and high positive (+3). This method is validated, considered better and more reliable when compared with the qualitative method, in which there is only visual analysis [33].

Serum was used for TNF-α quantification, which was performed according to Vendrame et al. [34], using the mouse TNF-α Quantikine immunoassay kit (R & D Systems # RTA00).

### Statistical analysis

Sample size was calculated according to the variation coefficient (10%) and the difference between the treatments considered significant (25%), with a probability of error of less than 5% and a power of 90%. The nonparametric Mann–Whitney test was used to compare groups for the continuity of adiposity index, adipose tissue weight, body weight, volume density, mean sectional area, total adipocyte volume, and relative expression of TNF-α, once these variables did not present normal distribution (Shapiro–Wilk test, *P*<0.05). Data for circulating TNF-α are presented as mean and standard deviation. Spearman’s correlation was performed to correlate weight, adiposity, and visceral fat weight variables. Data were analyzed, by a blinded researcher, using the IBM® SPSS® Statistics 22.0 program (Armonk, New York, U.S.A.). GraphPad Prism 5.0 (La Jolla, California, U.S.A.) was used to plot the graphs.

## Results

### Histological and stereological analysis of adipose tissue of rats after ingestion of the HGLI diet

**Figure 1:**
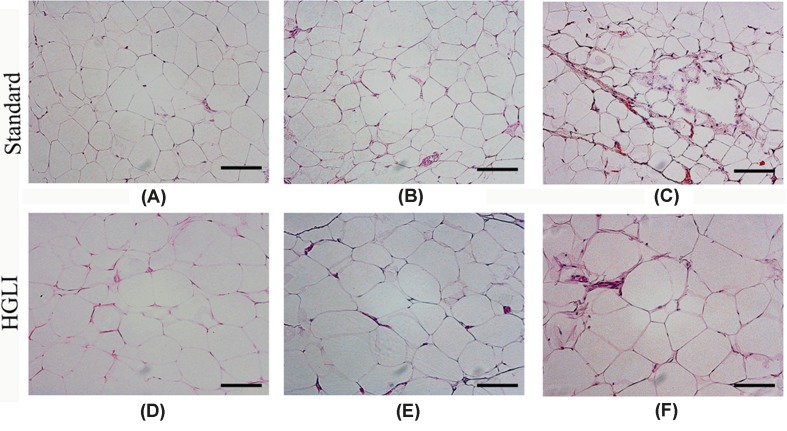
Photomicrography of adipose tissue of *Wistar* rats submitted to different treatments during 17 weeks All groups represent experiments with five animals. (**A**) Perirenal adipocytes in the standard group. (**B**) Retroperitoneal adipocytes in the standard group. (**C**) Epididymal adipocytes in the standard group. (**D**) Perirenal adipocytes in the HGLI group. (**E**) Retroperitoneal adipocytes in the HGLI group. (**F**) Epididymal adipocytes in the HGLI group; scale bars: 1000 µm. Magnification: 200× HGLI diet: mixture composed of Labina®, condensed milk and sugar (1:1:0.2). Standard diet (Labina® diet).

The presence of hypertrophy, coalescence, and plasma extravasation was diagnosed in the adipocytes of animals fed the HGLI diet, compared with animals fed a standard diet (Figure 1). In perirenal adipose tissue, we observed the presence of multilocular cells.

**Figure 2:**
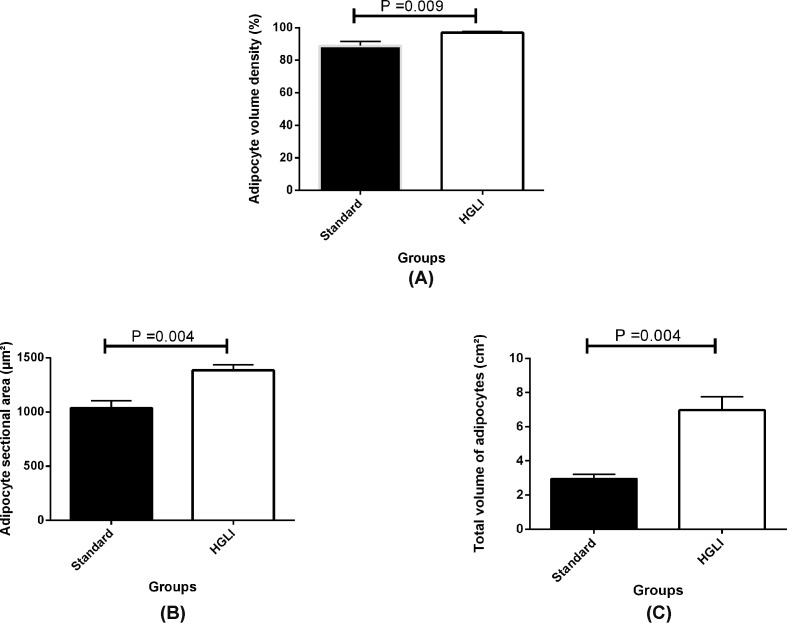
Stereology of adipose tissue of *Wistar* rats submitted to different treatments during 17 weeks All groups represent experiments with *n*=5 animals. (**A**) Adipocyte volume density in the standard and HGLI groups. (**B**) Adipocyte cross-sectional area in the standard and HGLI groups. (**C**) Total volume of adipocytes in the standard and HGLI groups. Values are presented as mean ± standard error and compared by the Mann–Whitney *U*-test. HGLI diet: mixture composed of Labina®, condensed milk and sugar (1:1:0.2). Standard diet (Labina® diet).

The animals fed the HGLI diet showed adipocyte volume density equal to 97.0% (*P*=0.009), cross-sectional area of adipocytes equivalent to 1387 μm^2^ (*P*=0.004), and a total volume of adipocytes of 6.97 cm^3^ (*P*=0.004), which corresponds to an increase in 8%, 25%, and 58%, respectively. This was significantly higher than the values observed to the animals consuming the standard diet (Figure 2), confirming the findings found in the histological analysis.

### Histological analysis of the small intestine of rats after the HGLI diet

**Figure 3:**
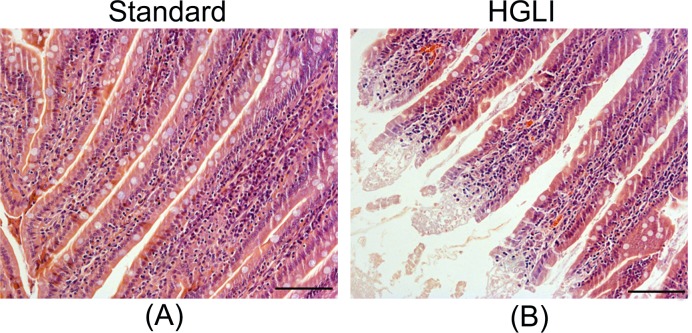
Photomicrography of jejunum of *Wistar* rats submitted to different treatments during 17 weeks All groups represent experiments with *n*=5 animals. (**A**) Jejunum in the standard group. (**B**) Jejunum in the HGLI group; scale bars: 1000 µm; magnification: 200×. HGLI diet: mixture composed of Labina®, condensed milk and sugar (1: 1: 0.2). Standard diet (Labina® diet).

The villi present in the central portion of the small intestine of the group fed the HGLI diet presented disorganization, with consequent epithelial dysfunctions due to the inflammation characterized by leukocyte migration to the apex of the villi (Figure 3).

### Liver and pancreas histological analysis after the HGLI diet

**Figure 4:**
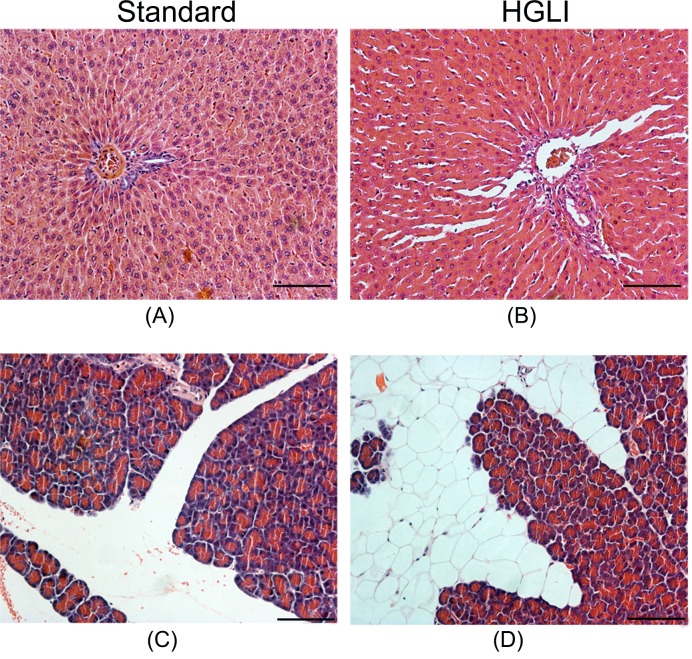
Photomicrography of liver and pancreas of *Wistar* rats submitted to different treatments during 17 weeks All groups represent experiments with *n*=5 animals. (**A**) Liver in the standard group. (**B**) Liver in the HGLI group. (**C**) Pancreas in the standard group. (**D**) Pancreas in the HGLI group; scale bars: 1000 µm; magnification: 200x. HGLI diet: mixture composed of Labina®®, condensed milk and sugar (1:1:0.2). Standard diet (Labina® diet).

Microscopic analysis showed that the liver of the animals that received the HGLI diet presented fat infiltration. There was an interlobular accumulation of adipocytes in the pancreas of the group of animals fed the experimental diet (Figure 4).

### Visceral adiposity in rats fed the HGLI diet

**Figure 5:**
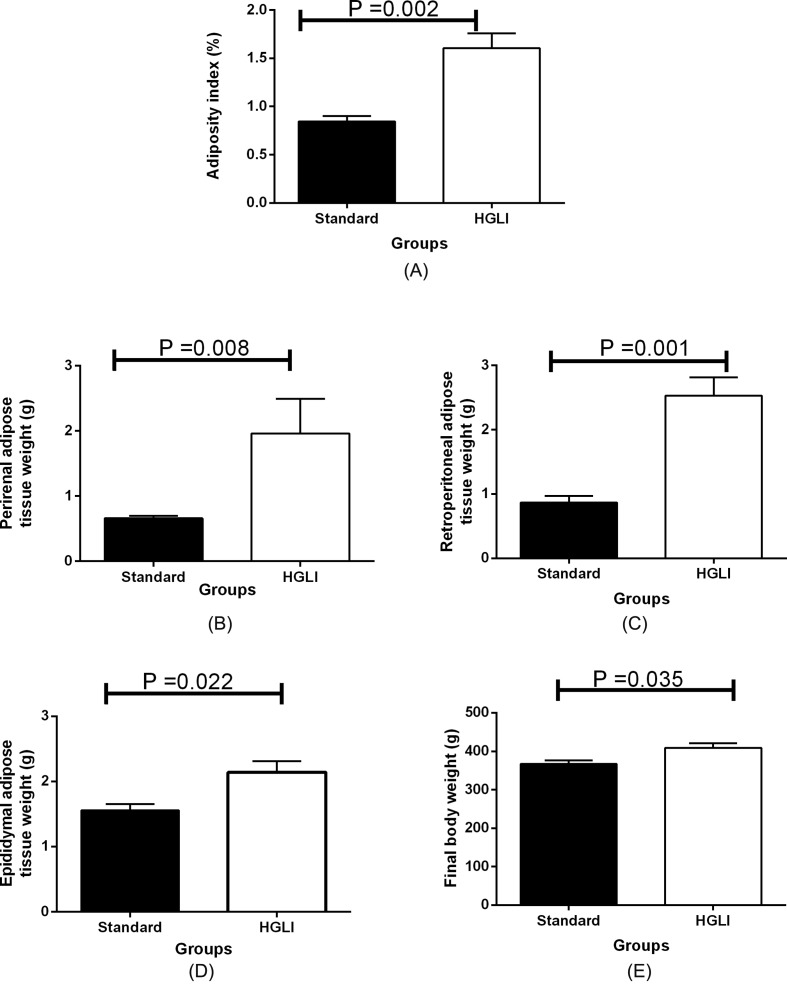
Visceral adiposity in *Wistar* rats submitted to different treatments during 17 weeks All groups represent experiments with *n*=5 animals. (**A**) Adiposity index in the standard and HGLI groups. (**B**) Perirenal adipose tissue weight in the standard and HGLI groups. (**C**) Retroperitoneal adipose tissue weight in the standard and HGLI groups. (**D**) Epididymal adipose tissue weight in the standard and HGLI groups. (**E**) Final body weight in the standard and HGLI groups. Values are presented as mean ± standard error and compared by the Mann–Whitney *U*-test. HGLI diet: mixture composed of Labina®, condensed milk and sugar (1:1:0.2). Standard diet (Labina® diet).

**Figure 6:**
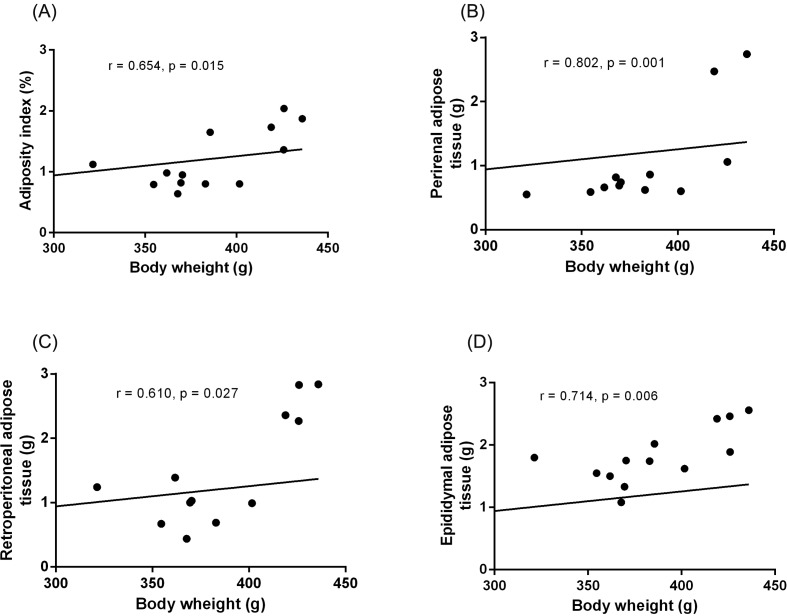
Correlation between body weight and adiposity index, perirenal adipose tissue, retroperitoneal adipose tissue and epididymal adipose tissue of *Wistar* rats submitted to different treatments during 17 weeks (**A**) adiposity index, (**B**) perirenal adipose tissue, (**C**) retroperitoneal adipose tissue, and (**D**) epididymal adipose tissue. All groups represent experiments with *n*=5 animals. HGLI diet: mixture composed of Labina®, condensed milk and sugar (1:1:0.2). Spearman’s correlation was used. Standard diet (Labina® diet).

Visceral adiposity index, the weight of each adipose tissue, and final body weight were higher in animals fed the HGLI diet (Figure 5). A positive correlation was observed between body weight and adiposity index (*r*=0.654, *P*=0.015). Body weight was also positively correlated with perirenal (*r*=0.802, *P*=0.001), retroperitoneal (*r*=0.610, *P*=0.027), and epididymal (*r*=0.714, *P*=0.006) adipose tissues (Figure 6).

### Relative expression of mRNA, immunohistochemistry, and plasma TNF-α concentration of animals after ingestion of the HGLI diet

**Figure 7:**
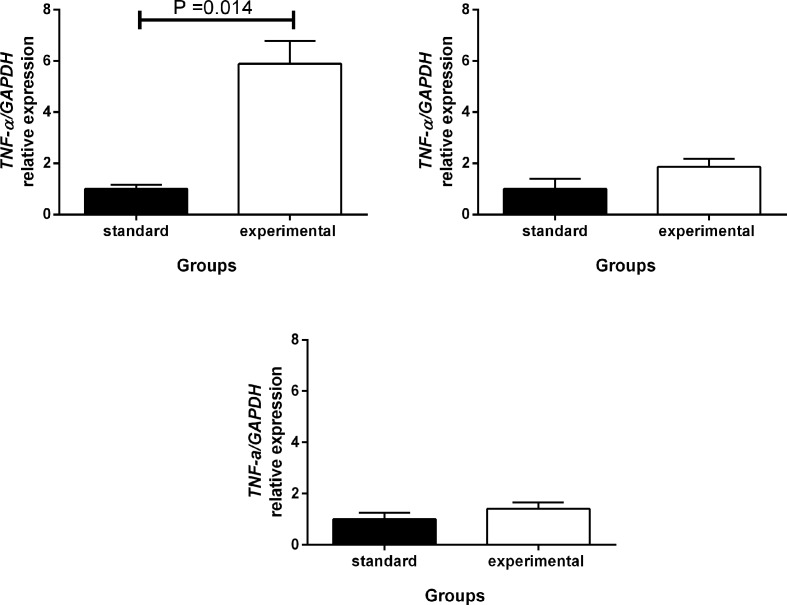
Relative mRNA expression of *TNF-α* in adipose tissue of *Wistar* rats submitted to different treatments during 17 weeks All groups represent experiments with *n*=5 animals. (**A**) Perirenal tissue in the standard and HGLI groups. (**B**) Retroperitoneal tissue in the standard and HGLI groups. (**C**) Epididymal tissue in the standard and HGLI groups. All data are expressed as fold change versus expression in the standard group, normalized to *GAPDH*. Values are presented as mean ± standard error and compared by the Mann–Whitney *U*-test. *GAPDH*: glyceraldehyde-3-phosphate dehydrogenase (Rn01775763_g1). *TNF-α*: factor de necrose tumoral alfa tumor necrosis fator-alpha (LOC103694, Rn01525859_g1). HGLI diet: mixture composed of Labina®, condensed milk and sugar (1:1:0.2). Standard diet (Labina® diet).

A significant increase in *TNF-α* (5.9-fold) mRNA expression in the perirenal adipose tissue (Figure 7A) was observed in the experimental group (HGLI diet), when compared with the group that received a nutritionally adequate diet (*P*=0.014). For the other tissues, it was observed that both retroperitoneal adipose tissue (Figure 7B) and epididymal adipose tissue (Figure 7C) did not show significant differences in *TNF-α* expression mRNA; however, animals fed the HGLI diet presented a slight increase in this expression than those receiving standard diet.

**Figure 8:**
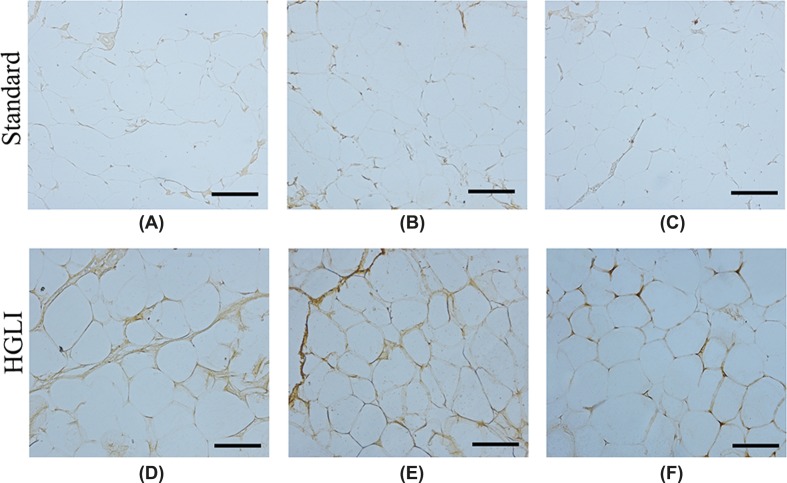
Immunostaining of TNF-α in adipose tissue of *Wistar* rats submitted to different treatments during 17 weeks All groups represent experiments with *n*=5 animals. (**A**) Perirenal adipocytes in the standard group. (**B**) Retroperitoneal adipocytes in the standard group. (**C**) Epididymal adipocytes in the standard group. (**D**) Perirenal adipocytes in the HGLI group. (**E**) Retroperitoneal adipocytes in the HGLI group. (**F**) Epididymal adipocytes in the HGLI group. Bars indicate 1000 µm. HGLI diet: mixture composed of Labina®, condensed milk and sugar (1: 1: 0.2). Standard diet (Labina® diet).

Immunohistochemistry showed a discrete immunostaining of TNF-α in the adipose tissue of control animals, unlike the HGLI group, which presented intense staining of this cytokine in all adipose tissue compartments evaluated (Figure 8), with positive immunostaining (+2), by optical density (Table 1).

**Table 1:** Score of TNF-α immunostaining in adipose tissue of *Wistar* rats submitted to different treatments during 17 weeks.

	Standard			HGLI*		
	Perirenal	Retroperitoneal	Epididymal	Perirenal	Retroperitoneal	Epididymal
**Negative (0)**	4	5	5	–	–	–
**Low positive (+1)**	1	–	–	1	1	2
**Positive (+2)**	–	–	–	4	4	3
**High positive (+3)**	–	–	–	–	–	–

All groups represent experiments with *n*=5 animals; TNF-α, tumor necrosis factor-α; ^*^HGLI diet: mixture composed of Labina®, condensed milk and sugar (1: 1: 0.2). Standard diet (Labina® diet).

Animals receiving the HGLI diet had a change in plasma TNF-α concentrations, which showed a mean (SD) of 6.4 (1.22) pg/ml, whereas values of all animals receiving the standard diet were lower than 5.0 pg/ml, which is the minimum detection value proposed by the method used. This shows that the HGLI diet was able to generate inflammation in *Wistar* rats.

## Discussion

Adipose tissue, besides being an endocrine organ, acts as a caloric reservoir, which under excessive conditions of food intake stores excess in the form of neutral lipids [35]. In situations of obesity, a disordered remodeling of this tissue occurs [36]. In thr present study, we identified hypertrophic, unilocular, and coalescence cells in animals receiving the HGLI diet. This hypertrophy is related to lower adipogenesis, the production of few adipocytes over time, generating a greater accumulation of lipids in existing adipocytes [37]. According to Reilly et al. [38], adipocytes expand to accommodate and store lipid augmentation due to the anabolic force of hyperinsulinemia, stimulated, especially, by foods with a high glycemic index and high glycemic load causing an increase in adipocyte stress.

In the present study, it was possible to prove such hypertrophy based on the stereological data, since there was a significant increase in adipocyte volume density, as well as the cross-sectional area and the total volume of these. It is also worth noting that hypertrophy of adipocytes in visceral adipose tissue is associated with dyslipidemia [39] and the study carried out by Aguiar et al. [25], with the same HGLI diet, showed elevated fasting glycemia, triglycerides, and very low density lipoprotein in the plasma of *Wistar* rats.

Additionally, the group of animals fed the standard diet presented multilocular cells in the adipose perirenal tissue, indicating the presence of brown adipose tissue [40]. This finding seems to be associated with the maintenance of energy balance in rodents [41]. Cinti [42] stated that white adipose tissue has a variable amount of brown adipocytes, which depends on factors such as age, species, environmental, and nutritional conditions. It should be noted that the animal groups in this experiment were distinguished only in terms of nutritional conditions, thus, these findings corroborate the literature, revealing that a high carbohydrate diet was able to cause white adipocyte pre-eminence, favoring an increase in lipogenesis [43], as opposed to the nutritionally adequate diet, which maintained brown adipocytes in the white area.

Regarding the adiposity index, as well as the weight of the different compartments of visceral adipose tissue and the final body weight, a significant increase in all these parameters was observed in the animals fed the HGLI diet compared with the group fed with the standard diet. Such data were confirmed by the positive correlation observed between them. Aguiar et al. [25], in their study had already observed obesity, using as a parameter, the Lee index, which resembles the body mass index used for humans, and an increase in waist circumference in animals consuming the HGLI diet. Changes in body composition and, consequently, remodeling of adipose tissue can lead to dysfunction [44,45], with alteration in the release of cytokines and inflammation markers such as TNF-α, interleukin-6, monocyte chemotactic protein, plasminogen activator inhibitor-1, and C-reactive protein [46]. This condition may also alter the production and signalization of protein hormones, such as leptin, visfatin, resistin, and adiponectin [47].

The alteration in the release of these proteins affects processes in the periphery and central nervous system [48], besides generating an ectopic accumulation of visceral fat in several organs [49,50]. Such accumulation has been associated with gastrointestinal complications, which may lead to acute pancreatitis, pancreatic cancer, non-alcoholic fatty liver disease (NAFLD), cirrhosis, hepatocellular carcinoma, inflammatory bowel diseases, as well as accelerated glucose intestinal absorption [51]. Obesity and chronic intestinal inflammation are already well documented in the literature [52–54], as well as their consequences in reducing insulin sensitivity and increased glucose uptake [55,56]. In the present study, it was possible to detect in the intestines of rats fed the HGLI pellet-diet rupture and dysfunctions of the epithelial layer with inflammation characterized by leukocyte migration to the apex of the villi.

Several studies have shown that alteration of the intestinal barrier, commonly found in individuals with obesity, is the main cause of endotoxemia, systemic, and liver inflammation present in obesity [52,57]. In this sense, TNF-α has received considerable attention due to its involvement in the regulation of the intestinal barrier function [58,59]. Thus, there is great interest in studies involving experimental obesity induced by diets, resembling dietary patterns of the occidental modern society, which may induce dysfunction in the intestinal barrier. Beverages with a high carbohydrate content, mainly fructose, have more easily caused damage to the intestinal epithelium, unlike strictly pellet-diets [16,60]. These negative changes in the intestinal barrier promote research involving the recovery of intestinal permeability and also the microbiota.

Volynets et al. [60] using pellet-diets supplemented with fructose-rich beverages in mice also observed that ingestion caused damage to the intestinal epithelium and consequently to the function of the intestinal barrier. It should be noted that in the present study there were alterations of the intestinal villi without the addition of beverages rich in fructose. A study using a Western-style diet in *Wistar* rats found edema in the villi, lymphocyte infiltration, and goblet cell hyperplasia in the ileum [61]. Prajapati et al. [62], using the same diet, observed destruction of the epithelial layer in the ileum of rats. However, these studies did not evaluate changes in the jejunum (intestinal portion responsible for the absorption of nutrients) nor parameters related to the presence of TNF-α in these animals. Besides, the diet used did not present a high glycemic index and high glycemic load.

In the liver, the fatty infiltration observed in animals fed the HGLI diet probably occurred due to the high fructose consumption, since the diet is rich in sucrose. This same diet had already caused dyslipidemia in a previous study [25]. According to studies by Wree et al. [63] and Ferreira et al. [43], there is evidence suggesting that fructose excess plays an important role in the progression of NAFLD since this carbohydrate is highly lipogenic. In the present study, the pancreas of the animals that consumed the HGLI diet showed infiltration of adipocytes and this finding has been related to obesity [64], which is associated with the metabolic syndrome [65] and the NAFLD [66,67].

Hepatic lipogenesis due to inadequate carbohydrate intake is also associated with increased release of inflammatory cytokines, such as TNF-α [68]. In the present study, a significant increase in the relative expression of *TNF-α* mRNA in the perirenal adipose tissue of the animals fed with the HGLI diet was identified. Furthermore, according to immunohistochemistry, animals fed the HGLI diet showed strong inflammatory characteristics, with intense TNF-α staining, and positive immunostaining when compared with animals receiving the standard diet. Finally, the present study identified high TNF-α plasma concentrations in the animals that consumed the HGLI diet, being an additional evidence of inflammation. It should be noted that these results in adult male *Wistar* rats submitted to a pellet-diet of a high glycemic index and high glycemic load have not been previously seen in the literature as far as we know.

In a comparative study between a diet rich in condensed milk and one rich in fat, Masi et al. [69] found increased body weight gain, glucose intolerance, hepatic fibrogenesis, increased relative expression of collagen mRNA and TNF-α in the liver, and leptin in epididymal adipose tissue of C57BL/6 male mice fed with the diet rich in condensed milk, indicating that this ingredient is more inflammatory than fat, represented by lard. The present study confirms the development of inflammation in *Wistar* rats caused by the HGLI diet, which has condensed milk as the main ingredient.

A study by Muralidhar et al. [19], evaluating the effect of a sucrose-rich pellet-diet, found that the diet was not able to raise body weight gain and visceral adiposity in *Wistar* rats, which showed mean adipocytes area comparable to those fed with the standard diet. In addition, circulating TNF-α and TNF-α in adipose tissue also did not change. This was not the case of the present study, which demonstrated that the HGLI pellet-diet was able to generate dysfunction in adipose tissue with evidence of chronic inflammation in *Wistar* animals.

Remodeling of adipose tissue in *Wistar* rats, represented in the present study by adipocyte hypertrophy, as well as changes in the intestinal epithelium, demonstrates an association with inflammation, evidenced by the increase in TNF-α plasma concentrations, increase in the relative mRNA expression of this cytokine in the perirenal adipose tissue and immunostaining in all of the adipose tissue compartments of the animals that consumed the HGLI diet. There are no records in the literature of such alterations with the use of this type of diet, especially when it refers to changes in the intestinal epithelium in a state of obesity, which are possibly related to the increase in TNF-α, as already well established in the literature [58,59]. Thus, our experimental model presents clinical applicability and may be used in the investigation of numerous dysfunctions associated with obesity and inflammation, aiming its treatment. One of the limitations of the present study is that only one inflammatory cytokine was evaluated. Analyzing other cytokines would have made the characterization of the relationships between lipogenesis–obesity–inflammation even clearer.

The results of the present study are consistent and coincide with studies conducted by other researchers who used high-carbohydrate diets [17,69], including Oliveira et al. [18] who used a similar diet to that of our research group. These studies verified that there was an increase in body weight and adipose tissue, with a consequent increase in inflammation. However, all these studies were conducted in mice, which are inbred strains [70]. In addition, these studies did not evaluate diets glycemic index and glycemic load, characterizing diets as being of high or low glycemic index based only on the type and/or percentage of starch components used [71–75]. In a recent meta-analysis, published by Campbell et al. [11], this information was highlighted as a limiting factor in experimental studies.

Studies on the disorders involving obesity are still needed to understand its molecular basis and improve treatment. Experimental models are promising, once easily reproduced. This is the first low-cost pellet-diet with a high glycemic index and load, efficient in causing obesity, increased waist circumference, changes in biochemical parameters, and elevation of PPAR-γ expression in *Wistar* rats [25]. When compared with the already existing high-fat diets, the HGLI diet should be chosen as an effective tool in research, once inflammatory dysfunctions are presented in *Wistar* rats, unlike high-fat diets [12]. Furthermore, the HGLI diet has condensed milk as the main ingredient, which is more inflammatory than fat [69].

## Conclusion

The present study demonstrates that this diet was able to increase visceral adipose tissue, based on morphological and stereological parameters, promote ectopic accumulation of fat in the pancreas and liver, as well as inflammation in the intestinal epithelium and increased TNF-α. Therefore, the HGLI pellet-diet may be considered an effective tool to be used by the scientific community in experimental research.

## Abbreviations

DIO: diet-induced obesity
HGLI: high glycemic index and high glycemic load
NAFLD: non-alcoholic fatty liver disease
PPAR-γ: Peroxixome Proliferator-activated receptor-γ
TNF-α: tumor necrosis factor-α
